# Clinicians in management: a qualitative study of managers’ use of influence strategies in hospitals

**DOI:** 10.1186/1472-6963-14-251

**Published:** 2014-06-13

**Authors:** Ivan Spehar, Jan C Frich, Lars Erik Kjekshus

**Affiliations:** 1 Department of Health Management and Health Economics, Institute of Health and Society, University of Oslo, P.O. Box 1089, Oslo NO-0318, Norway

**Keywords:** Norway, Professions, Power, Roles, Managers, Health care, Doctor, Nurse

## Abstract

**Background:**

Combining a professional and managerial role can be challenging for doctors and nurses. We aimed to explore influence strategies used by doctors and nurses who are managers in hospitals with a model of unitary and profession neutral management at all levels.

**Methods:**

We did a study based on data from interviews and observations of 30 managers with a clinical background in Norwegian hospitals.

**Results:**

Managers with a nursing background argued that medical doctors could more easily gain support for their views. Nurses reported deliberately not disclosing their professional background, and could use a doctor as their agent to achieve a strategic advantage. Doctors believed that they had to use their power as experts to influence peers. Doctors attempted to be medical role models, while nurses spoke of being a role model in more general terms. Managers who were not able to influence the system directly found informal workarounds. We did not identify horizontal strategies in the observations and accounts given by the managers in our study.

**Conclusions:**

Managers’ professional background may be both a resource and constraint, and also determine the influence strategies they use. Professional roles and influence strategies should be a theme in leadership development programs for health professionals.

## Background

There has been an increased emphasis on engaging clinicians into management [1]. While the focus on efficiency, effectiveness and quality of care has played a role in public discourse, others point to the engagement of clinicians being critical to successful healthcare reforms [2]. There have also been attempts to co-opt clinicians into management roles in response to the shortcomings of New Public Management and professional resistance towards top-down initiatives and directives [3-5]. Researchers have argued that policy makers fail to understand professional social structures that could threaten the effectiveness of policy drives and management reforms designed to engage clinicians in management [6,7]. Resistance to change and conflicts in health care organizations may be rooted in power struggles and the organizational structure. There has been little research on the ways in which managers with a clinical background exercise influence. Understanding more about the factors that determine their influence strategies may be important for training and support.

In Norway, a new law required unitary management at all levels in hospitals from 2001 [8]. Previously, hospitals had been run by doctors and nurses in two parallel hierarchies. Unitary and “profession neutral” management was enforced to strengthen accountability and professionalize management. Managers became responsible for all employees in a department, and a manager with a nursing background would be managing the doctors in a department and vice versa. The model departs from governance models commonly used in other countries, where the main responsibility for running clinical departments usually lies with a doctor, either alone or together with a general manager and a nurse [9]. The Norwegian case represents a unique opportunity to study the variations in influence strategies used by managers with a clinical background.

### Aim of the study

We did a study to explore influence strategies used by doctors and nurses who are managers in hospitals with a model of unitary and profession neutral management at all levels.

### Theoretical perspectives

#### Hybrid management

The terms “hybrid leadership” and “hybrid management” have been used to describe managers who combine a professional background with managerial skills and responsibilities [5,10]. Within healthcare, the term “hybrid” reflects an underlying assumption that medicine and management represent two different logics, and that a hybrid manager is able to embody, translate and mediate between the logics of management and medicine [5,11-14]. The term is used to refer to doctors [10], but has also been used to describe nurses and other professionals [15,16]. Savage and Scott [17] have defined hybrid management as “a new type of management in which non-medical health care professionals engage in aspects of general (or ‘generic’) management, combining this with their clinical management responsibilities”. While there are national differences in how clinicians have reacted to top-down initiatives, new hybrid roles have appeared in several countries, including Denmark, Finland, England and Australia [4,14,18].

In this study, we focus on clinicians in formal management positions who may or may not retain a role in clinical work. These managers could also be described as “hybrids”, as they combine a professional background with a formal position in management.

#### Influence and power

Power may be defined as “the ability to affect others’ beliefs, attitudes, and courses of action” [19]. Hospitals are sites for continuous exercise of influence and power, including competition over resources, jurisdiction, tasks and mindsets [20-22]. The language of “battles” and “fights” has been especially apparent in the sociological literature, such as in the work of Abbott [20] on the system of professions and Freidson's [23] work on professionalism and professionals’ claims of expertise. The literature on hybridity reflects these struggles, and Waring and Currie [24] have shown how managerial expertise can be detached from managers and drawn into professional practice, enabling professionals to extend their influence over management and avoid unwanted interference in their work.

Mintzberg [25] has described hospitals as “professional bureaucracies” in which power resides in expertise through knowledge and skills. These organizations are characterized by an inverted power structure, where front-line staff usually has more influence over daily decision making than those in formal positions of authority. Managers need to acknowledge this culture when negotiating with staff [26]. Braithwaite and colleagues [27] assert that their jobs “are more about negotiation and persuasion than command and control”. French and Raven have published a typology of various power bases [28]: legitimate (having a formal position or title), reward (ability to compensate another for compliance), expert (superior skills, experience and knowledge), referent (perceived attractiveness) and coercive (ability to punish others for non-compliance). Informational power (potential to utilize information) was later added as a sixth power base [29]. Building on French and Raven’s [28] framework, Northouse [19] distinguishes between position power, the power an individual derives from a position or status that embodies notions of legitimate, reward and coercive power, and personal power that embodies the notions of referent and expert power [19]. We hypothesized that a hybrid managers’ professional background could have an impact on what power bases they had access to.

#### Role as resource

Roles are often defined as the behavioral expectations associated with and emerging from positions in a social structure [30]. Usually, structures will be a constraining feature of social roles, while interactionist perspectives highlight independence and agency in role-playing [31]. The theory of role as resource is an example of an agent-centered perspective. Baker and Faulkner [32] found that filmmakers used different roles, such as producer and screenwriter, strategically to gain legitimacy, underscoring that roles can be used as platforms for exercising power and influence. Callero [33] followed up on this idea, arguing that roles, being cultural constructs, could both facilitate and constrain. Roles enable access to cultural, material and social resources, and an individual in a given role can exploit these to pursue personal or group interests. Firstly, a minimum level of cultural endorsement or acceptance needs to exist for a role to be used as a resource. Stronger acceptance of the role increases its accessibility as a resource. Secondly, Callero [33] makes an analytical distinction between cultural endorsement and cultural evaluation. Although a role might be recognized and perceived as legitimate, it can simultaneously be evaluated in a negative light. Thirdly, roles with high prestige become more effective tools for gaining power. Callero argues that these types of roles tend to require long-term education (specifically mentioning doctors as an example), be severely limited in number, or require a highly valued commitment to the role.

Hybrid managers combine a professional background with a formal position or status as manager [1], and they often move in and out of roles [34]. The focal point of interest in our study is the manager’s role as doctor or nurse. Doctors generally hold a high social and cultural position within society [35]. Compared to nurses, doctors have higher income, longer education and more professional autonomy. Both professions have high degree of cultural endorsement, but evaluation and prestige is usually lower for nurses, a pattern seen in society in general, as well as in hospitals [36]. We believe that the differences in status may have an impact on how they use their professional role as a resource. We anticipated that there may be differences in their access to power, and, consequently, what strategies they use to exert influence. Viitanen and Konu [37] studied the leadership roles that were used by middle managers in Finnish health organizations, and nurses more often took on a mentor and facilitator role compared to doctors, who were more task-oriented. Furthermore, hybrid managers are located in-between a managerial and clinical mindset. While the former emphasizes a hierarchical approach towards power and influence, the latter emphasizes decentralized decision making [38]. We therefore expected that managers’ influence strategies vary according to whether they seek to exert influence upwards (towards a managerial mindset) or downwards in the hierarchy (towards a clinical mindset).

## Methods

### Ethical approval

Ethical approval to conduct the study was granted by the Norwegian Social Science Data Services (ref: 23228/2/LT). Written consent to participate in the study was obtained from all of the study participants.

### Setting and participants

Considering the explorative approach of this paper, we found a qualitative approach appropriate, and we did a study collecting data through individual interviews and observations of 30 managers with a clinical background. Norwegian public hospitals are organized in local health trusts, which could consist of several hospitals, and four regional health authorities. Our participants spanned across four public hospitals in two health trusts. One health trust had a five-level hierarchical management structure, consisting of the executive director of the organization, division managers, department managers, section managers and unit managers. The other had a four-level structure, excluding the unit management level but otherwise similar. The first author contacted division and department managers and asked for permission to contact potential participants directly through email and phone. In a few cases the superior forwarded our request directly to the participants, who then contacted the first author. We used a maximum variation sampling strategy in order to include a wide range of informants with a broad array of experiences. We sought variation in terms of hospital size (university hospital or local hospital), clinical specialty (internal medicine or surgery), management level (department or section) professional background and gender. The sample includes 16 nurses, 13 doctors and a participant with another health care background. Characteristics of the participants are presented in Table 1. We recruited 20 managers from medical departments and 10 from surgical departments.

**Table 1:** Characteristics of participants (N = 30)

**Characteristics**	**No**	**%**
**Gender**		
Female	17	(57)
Male	13	(43)
**Age**		
36-45	9	(30)
46-55	12	(40)
56-65	9	(30)
**Management level**		
Department	17	(57)
Section (includes nine first-line managers)	13	(43)
**Mean age**		
Doctors	55	
Nurses	49	
Other clinical background	40	

### Data collection procedure

The interview guide was developed on the basis of theoretical studies and was revised based on data from two pilot focus group interviews with 20 clinicians in an executive program in health administration, and who did not participate in any of the subsequent interviews or observations. The first author, a doctoral student with a background in psychology, conducted tape-recorded, face to face in-depth interviews with all 30 participants at their workplace. None of these 30 had participated in the focus group interviews. The interviews lasted from 45 to 90 minutes. The first author also observed 20 of the participants (11 department managers, 9 section managers) in staff and management meetings and during informal talks with colleagues. The data was collected from March to December 2010. By combining interviews with observations, we were able to look for consistency and discrepancies in the stories that participants told, and gain more insight into how they were “doing” management. Observations were also important for understanding the actors’ perceptions and interpretation of their own social world and generating independent insight into the organizational structures and work life. Observations were carried out on the same day as the interviews, and IS usually met up with the participants at the start of their work day and followed the participant throughout the day. The authors did not participate in clinical consultations with patients. Observations were documented in field journals and kept for later analysis.

### Data analysis

Several steps were taken to ensure the quality of the analysis. We used NVivo8 computer software to facilitate the analysis of interview transcripts and field notes. The interviews were analyzed by systemic text condensation [39]. This approach followed four steps: (1) Reading all of the material to form an overall impression; (2) identifying units of meaning and subsequently coding for these units; (3) condensing and summarizing the contents of each coded group; and (4) generalizing the description and contents reflecting participants’ attempts at exerting influence. Transcripts of several of the interviews were analyzed for content and structure by all three authors, resulting in general agreement on a coding frame. Field notes were analyzed independently for emerging themes and then assessed against findings from interviews, with special interest on observations that could validate, contradict or add additional insights to the interview data.

For the purpose of methodological and analytical clarity, we chose to focus the analysis mainly on accounts given by managers at the department level because they have similar assignments and responsibilities regardless of clinical background. While nurses usually have responsibility for a larger number of staff and allocate most of their time to staffing and scheduling shifts, doctors are able to allocate more of their time to medical and academic work. Experiences from interviews and observations of section managers have in some cases been included when relevant to our study aims. This includes section managers who had previous experience as department managers, or who recounted encounters with other department managers.

## Results

Managers with a nursing background argued that medical doctors could more easily gain support for their views. Nurses reported deliberately not disclosing their professional background, and could use a doctor as their agent to achieve a strategic advantage. Doctors believed that they had to use their power as experts to influence peers. Doctors attempted to be medical role models, while nurses spoke of being a role model in more general terms. Managers who were not able to influence the system directly found informal workarounds. We did not identify horizontal strategies in the observations and accounts given by the managers in our study.

We have organized the results in two sections: the strategies that managers used to influence upwards in the management hierarchy (towards their supervisor and top management), and the strategies they used to influence downwards in the organization (towards section managers and the professional staff). Table 2 summarizes the variety of strategies used by the managers in our study, and how they relate to different bases of power. We describe these in detail below.

**Table 2:** **Examples of the variation in influence strategies used by managers in our study, and how they relate to different bases of power**[28]

**Influence strategy**	**Type of power**
**Upwards in the hierarchy**	
Advance professional considerations/concerns	Expert
Use a doctor as one’s agent to increase argumentative strength	Expert
Use different titles strategically	Expert
“Whine”/argue that “everybody else gets more resources”	Informational
Avoid shouting “wolf” too often	Informational
Sabotage	Coercive
**Downwards in the hierarchy**	
- Be a professional role model (e.g. performing surgery)	Expert
- Challenge arguments (e.g. “I have done this procedure before”)	Expert
- Be a general role model (e.g. arriving early to work)	Referent
- Be a facilitator (e.g. doing the “crappy” work)	Referent
- Rephrase and redefine language	Informational

### Influencing upwards

Participants told that they attempted to emphasize their employees’ competence when arguing upwards in the organization. They believed that they had to present professional arguments in order to be heard, but also expressed distrust towards the higher level managers, feeling ignored or being misunderstood. A department manager with a medical background told how he had rearranged his working day to make a point:

The management thinks that [our department] hospitalizes too many patients, based on some numbers from a few years ago. We hospitalize more patients than another hospital in our health trust. The other hospital sends them to another hospital and never sees them. It’s a very complicated and expensive patient group, the other hospital doesn’t have that at all, while it’s a large part of our activities. […] And those numbers don’t take into account the travel distance. Some patients travel three hours to get here. Elderly, complicated illnesses. Are we going to send them home again with a taxi? That’s expensive.

[They said] “we have to do something about it”. And then I said: “Ok, everything will go through me. No one can hospitalize a patient to this department without going through me”. I did this for several weeks. It didn’t really influence the number of admissions.

Interviewer: Did you communicate this to the top management?

Yes, but it isn’t so easy to, you know, what we are talking about now are excuses. The figures [emphasized by the participant] are there. They deal with the figures. And then we have to show that we are doing something about it. And what we do is that I deal with these admissions myself.

The participant spoke of this as a form of “sabotage”, as a way of making his concerns visible and being heard upwards, even though it prevented him from performing his other managerial tasks: “It has influenced my work day to the point where it has become almost impossible”. It was important for participants that their superiors understood their work and the challenges they had to manage. The manager took on these tasks because he experienced that the management was only interested in figures, without asking what was behind them. This was a frequent concern, and utterances such as “the management level above doesn’t know our day-to-day reality” were frequent. In cases where department managers experienced that they were not able to persuade higher level managers through professional or logical arguments, they found ways of sabotaging or circumventing the system, as illustrated above. There were also other examples of workarounds and sabotage. A department manager spoke of circumventing the system by calling IT-support directly on their mobile phone. This was faster and more efficient than going through the formal system for contacting technical support:

We have people we can use when we understand how to circumvent the system. We have their private mobile phone numbers and can call them unofficially and say “you have to help me”. “Well, I’m not allowed, but I’ll come”. We make it work that way. But it’s absolutely unofficial and illegal. Because it’s not supposed to be like that. And they get reprimanded if they help us, unless it’s through the service phone or the helpdesk phone.

During one of the observations, a department manager with a medical background suggested to one of his section managers that they could buy modern, experimental equipment, and when other necessary equipment would be broken, the health trust would have to replace that equipment. This would be a way of ensuring additional medical equipment, without having to use the existing money on upgrading old equipment.

Budgets were described as something one had to “fight” for. A manager with a nursing background told that she had managed to “whine” herself into acquiring a new member of staff, by saying “why should the other [departments] have more staff than us, when we have just as much to do”. Another participant spoke of the importance of not complaining or shouting “wolf” too often, in order to be taken seriously by one’s supervisor.

Nurses in section management positions spoke of benefits of having department managers with a medical background, because their department would stand stronger in negotiations for budgets and resource allocations:

…and then you can say that in some battles it would be an advantage or disadvantage if my supervisor was a nurse or doctor, you know, will the nurse be as strong in all situations and discussions as if the person had been a doctor instead. Because the doctors have strong credibility in the system.

Some managers with a nursing background used their medical advisors strategically to carry their own agendas across. An example is provided below:

And it happens sometimes, when I’m arguing for certain issues, when I’m going into discussions with groups of doctors […] I may consciously use the senior consultant to strategically front my views. There are some that think, or at least I think that a part of the system regards your arguments as weak when you don’t have that medical background, unfortunately. So I sometimes push the head senior consultant [doctor] strategically in front of me to win through (department manager with a nursing background).

A section manager with a nursing background told that she had sometimes used her job title strategically. Following changes in organizational titles, her title had changed from “department nurse” to “section manager”. Although she still used her old title, because she liked the connection to her profession, she made sure to change from the less powerful “department nurse” title to the more ambiguous “section manager” to gain leverage in strategic situations:

What I have sometimes used it for, the section manager title, is related to authority, it gives a little more authority to say that you are a section manager, I’ve experienced. For example, if we have to contact the chief district doctor. I’ve noticed that another title can be useful in those circumstances. Then you get more impact. I’m also a deputy for the department manager, so I’ve used that as well. I’ve had a sign where it said “deputy for the department manager”.

### Influencing downwards

A recurrent theme in the accounts given by the doctors, was the importance of being perceived as a competent clinician in order to be taken seriously by the medical staff. A surgeon described it with the following example:

If a non-doctor attempts to take medical decisions it usually goes wrong. Not because the decisions are bad, but because they don’t get support from below… and that’s why it has been important for me to demonstrate that, yes, I am doctor, yes, I understand what we do and yes, I can contribute. And that’s why I also made a point of going in and doing a complicated procedure, because nobody else were able to do it, because the guy who was supposed to do it was ill, and a patient coming from a city [1,600 kilometers away in distance] would have to be sent home. And then I did it, even if it messed up my day. Because it gives, you know, afterwards people talk about it and say “yeah, at least he is able to contribute and work”, and that gives respect among surgeons.

Another influence strategy mentioned by participants with a medical background was to become good at a particular niche in their professional field. This served as a form of compensation for clinicians who had to cut back on the time spent in the clinic, because of increasing management responsibilities. “One strategy is to become very good at one specific thing, for example pacemakers. The doctors will say: ‘well, he can’t really do that much surgery, but he is really good with pacemakers’” (a department manager working within a surgical department).

Nurses were more concerned with profession neutral ways of appearing as role models. In the example below, a department manager with a nursing background told of the importance of arriving on time for meetings:

The attitude one radiates, it influences, like expectations, you know. For example, when we meet in the morning at eight for joint meetings and when someone holds a lecture like today, then I think it’s rude to arrive five or seven minutes late. It interrupts and it’s impolite towards the person who has spent hours to prepare the presentation. It’s obvious that if I come dragging myself in five six seven minutes late every day or every other day, it will give signals. That these things are ok to do.

Although nurses told that they were proud of their nursing background, they appeared to downplay their professional background, emphasizing instead their role as facilitators or someone who took care of the “crappy things” for the doctors. One of the department managers with a nursing background told that she was challenged by doctors on how they would be able to do clinical research in the department:

When I began as a manager and was a nurse, then you hear that thing about “how are we going to do research in our department?” I say that I will facilitate so that you can conduct research. I will take all those crappy things outside, the practical things, you won’t have to sit and talk with all these people about whether you need to fill out this or that form. […] They won’t need to have to do all that. I think that’s really important for the doctors, that they feel that someone can take all of those things and that they can do their own things. Facilitating, enabling them to do it.

Participants also spoke of the importance of having worked among front-line staff. A participant with a medical background commented:

It’s worth its weight in gold that I have worked on the floor, then I know as a manager how things work, and what’s realistic and can say “it’s not like you say”. How can you prioritize between all the demands from the different section managers, if you don’t know what goes on in the department and how useful the different devices are?

Observations of participants in meetings and in discussions with staff provided examples of how their professional knowledge and experience became relevant when confronted by staff. In one situation, a department manager with a nursing background “won” a dispute with a section manager (also with a nursing background), because the former had previous experience with a specific intervention that they were discussing. The section manager tried to argue against the current organizing of syringes in relation to the intervention, to which the department manager disagreed. The department manager effectively ended the dispute by stating: “I have done those interventions myself”. While nurses generally appeared to downplay their professional background in negotiations with medical staff, this example illustrates that they could still use their nursing background strategically to “win” arguments against other nurses.

Participants did not only rely exclusively on their professional skills and experience in negotiations with staff. Observations of the participants also showed that both doctors and nurses engaged in rephrasing. A department manager was observed in a meeting with his section managers. He explained that the hospital would only get one new anesthetic machine, but the section managers replied: “We need more”. The department manager attempted to calm the situation by saying: “If we are going to have a ‘who has it the worst’, then [another hospital in the health trust] has it the worst”. “We need more” was in this way redefined to “others have it worse”.

## Discussion

### Role as both a resource and restraint

In this study, we were interested in exploring hybrid managers’ use of influence strategies and power, trying to differentiate between influence strategies used upwards and downwards in the organization. Our data illustrate how a professional background may both be a door opener and a restraint for action in both directions in the hierarchy. Callero [33] has argued that when roles serve as resources, behavior may be limited and constrained because one is being denied access to other roles. Our findings are in line with this observation. Not having a medical background, nurses believed that their impact upwards in the organization was not as strong as that of doctors, and they found other ways of accessing expert power. Nurses could draw indirectly on expert power by “disguising” themselves as doctors, or by using doctors as their agents to gain strategic leverage. A different pattern emerged in the influence strategies employed downwards in the organizations. As pointed out by Currie [6], because of the medical hegemony in decision-making, nurses’ influence over doctors is significantly reduced. We found that managers with a nursing background were able to draw on other types of power to achieve influence downwards in the organization. Nurses tried to be perceived as facilitators, by taking on administrative chores, thus shifting towards a referent power base.

While nurses were mostly restrained from acting on an expert base, a recurrent theme from interviews and observations of doctors was that they could not act *without* drawing on expert power. This was especially evident in the way that they sought to influence professional colleagues, which coincides with the expectations doctors have of professionals in management positions as the best among equals [40]. There appears to be a belief that simply having a medical background is insufficient for influencing medical colleagues. While a doctor might use expert power upwards in the hierarchy by virtue of being a doctor, in the same way as nurses might use a doctor as their agent, doctors believe that they have to maintain their clinical skills in order to retain credibility among peers e.g. [41,42]. Expert power is thus not earned once and for all, but had to be continuously reestablished and negotiated, which may represent a dilemma for doctors. For example, if a doctor relied on position or referent power in managing clinical staff, the doctors’ access to the expert base could be weakened over time as her or his status as an expert dwindled. Our findings suggest that roles do not serve to restrict behavior only because they constrain access to other roles (e.g. nurses being denied the role of doctor), but also because of the inherent expectations towards the role holder (e.g. doctors in leadership positions being perceived as the best among equals).

### Roles, power and influence in a hospital setting

Our results reflect the authoritative coordination mechanisms found in hospital settings, and how managers within this setting are influenced by those mechanisms. Although our participants had some freedom in choosing influence strategies, the strategies seemed to be determined by the power bases they could access. More specifically, the emphasis that the participants placed on expert knowledge limited the influence strategies that were available. While some power bases, such as expert knowledge, are not exclusive to healthcare organizations (they area also relevant in other professional bureaucracies, such as in universities and law and accounting firms), they reflect some of the institutionalized rules and norms that exist in a healthcare context, i.e. that power lies in expertise [43]. Legitimate power, understood as formal authority, appeared to be less visible in the strategies used by managers in our study. Clinicians, and especially doctors, might perceive an experienced or merited doctor to have the legitimate right to influence them. This could explain why hybrid managers tend to draw on personal power, rather than position power in dealing with clinical staff [19]. Position power is not very effective upwards in the hierarchy either, as a manager at the department level is placed below in the formal hierarchy. Thus, not using position power reflects the separate worlds in hospitals [44], where managers simultaneously inhibit the world of the formal management hierarchy and an informal, meritocracy based world. Position power seems not to be very effective in either.

Numerato and colleagues [3] did a comprehensive review and argued that the dynamics and interplay between management and professionalism could be classified in five ideal outcome categories: (1) managerial hegemony; (2) co-optation; (3) negotiation; (4) strategic adaptation; and (5) professional resistance. Hybridization occurs in between hegemony and resistance, through the merging of managerial and professional skills, values, tools and knowledge. In our study, managerial hegemony and professional resistance was demonstrated through doctors sabotaging or circumventing the system in response to “unyielding” managers. Our results do not demonstrate examples of professionals taking on managerial or bureaucratic tools and logics. Adaptation was instead demonstrated by the nurse who used a doctor as an agent and the nurse who used different job titles strategically. This reflects Noordegraaf’s [45] description of healthcare organizations becoming “ambiguous domains” in which expertise can no longer be isolated from other experts [45].

### Where are the horizontal strategies?

Our study suggests that clinicians might resort to using sabotage or finding informal and “illegal” workarounds. Although these influence strategies do not necessarily constitute a conscious attempt to punish top management, they have a coercive element, threatening to punish the whole organization. Further, these reactions appear more individualistic than collectivistic in nature. Indeed, a somewhat surprising finding was that we found no examples of horizontal strategies in the interviews and observations of the managers in our study. Participants appeared to be concerned mainly with their own department or professional sub-discipline, and more often spoke of other departments or hospitals in terms of “competitors” rather than “collaborators”.

Johnson [46] argued that coalition building, in the sense of gathering influential people together, plays a vital part in building power and influence. Ganz [47] tells the story of grape workers’ ability to mobilize support from other communities through building horizontal coalitions. A similar influence strategy in a hospital setting would be to mobilize support from peer department managers, but this strategy was not present in our data. One explanation may be that that coalition building fails when managers are too focused on their own functional silos [46]. It should be noted that a number of Norwegian hospitals have organized doctors and nurses in separate units following the implementation of unitary management, so that managers at the lower levels of the organization only manage their own professional group. For example, a study of Norwegian health trusts in 2009 revealed that 60% of all hospitals had separated the bed units as independent units with their own management [48]. Edmonstone [11] underscores that clinicians are trained to think on a micro-level, with clinical leaders having a micro-view focus on patients and patient service. In a sense, professionals become competitors and representatives for their own professional unit.

### Practical implications

Various authors have pointed out that policy makers fail to understand the social structures that exist in professionalized contexts [6,7,49,50]. The results of our study could inform policy making in this area. Our study highlights some of the institutionalized rules and norms that exist in hospitals, namely the perception that power lies in expertise and that managers with a clinical background are more likely to draw on expert power than on formal position power. While nurses are restricted from directly accessing expert power, doctors are in a sense also restricted - not from accessing expert power, but from avoiding to do so - because of the importance they place on being perceived as professional role models. Decision makers and top managers need to acknowledge the social structure in hospitals and the challenges facing managers with different backgrounds, before implementing new management models and responsibilities. Our study suggests that professional roles and influence strategies should be a theme in leadership development programs for health professionals.

### Methodological considerations and further research

Witman and colleagues [42] point to a systematic bias in the literature on managers in healthcare, in that most of the research is based on interviews, with little emphasis on the use of observations. By using observations, a researcher can generate a partially independent view of the experiences that respondents draw on to construct their realities [51]. The fact that we were able to observe participants throughout their work day gave us an opportunity to produce a greater pool of data and to observe possible discrepancies between what our informants said and did. Observational data confirmed and provided additional examples of themes that emerged from interviews. Another strength of our study is that we explored both doctors and nurses’ views and experiences in the same organizations. A limitation of our study is the high proportion of male doctors and female nurses. It would be ideal to have more variation in terms of gender and professional background. We asked participants about their perception of the role of gender in relation to management and power, and they did not perceive it to be important. We believe that our results are transferable outside of the Norwegian context, as professional hegemonies are common in hospitals and other health care organizations [6] and access to power is therefore likely to follow from one’s professional background, regardless of national context. We have also answered Baker and Faulkner’s [32] request for the utility of the theory to be explored by applying it to more complex organizations. We applied the theory to a professionalized context and developed it further by combining it with literature on hybrid managers and power.

Future studies could investigate our findings further, for example by addressing the access to and use of power bases by general managers in health care organizations. It would also be interesting to investigate the conditions under which horizontal strategies are more and less likely to be used. Lastly, we found examples of managers circumventing and sabotaging the system. Although we deemed it beyond the scope of our paper to discuss these findings in more detail, we encourage other authors to take on a more comprehensive study of these phenomena in hospitals. Possible research questions include in what ways the formal organization of hospitals promote the use of these strategies, and whether hospitals (and other health care organizations) could be organized so that strategies which are useful for the individual are also useful for the organization.

## Conclusions

Managers’ professional background may be both a resource and constraint and determine the influence strategies they use. Professional roles and influence strategies should be a theme in leadership development programs for health professionals.

## Competing interests

The authors declare that they have no competing interests.

## Authors’ contributions

All authors were involved in the design of the project. IS carried out the observations and interviews. JCF and LEK provided assistance with coding and analyzing data from the interviews. The drafts of this article were revised critically by all authors. All authors have approved the final version of the manuscript.

## Pre-publication history

The pre-publication history for this paper can be accessed here:

http://www.biomedcentral.com/1472-6963/14/251/prepub
